# Does the utilization of dental services associate with masticatory performance in a Japanese urban population?: the Suita study

**DOI:** 10.1002/cre2.10

**Published:** 2015-10-26

**Authors:** Miki Kikui, Takahiro Ono, Momoyo Kida, Takayuki Kosaka, Masaaki Yamamoto, Yoko Yoshimuta, Sakae Yasui, Takashi Nokubi, Yoshinobu Maeda, Yoshihiro Kokubo, Makoto Watanabe, Yoshihiro Miyamoto

**Affiliations:** ^1^ Department of Prosthodontics, Gerodontology and Oral Rehabilitation Osaka University Graduate School of Dentistry 1‐8 Yamada‐Oka Suita Osaka 565‐0871 Japan; ^2^ Division of Comprehensive Prosthodontics Niigata University Graduate School of Medical and Dental Sciences 8050, Ikarashi 2‐no‐cho Niigata 950‐2181 Japan; ^3^ Osaka University 1‐1 Yamadaoka Suita Osaka 565‐0871 Japan; ^4^ Department of Preventive Cardiology National Cerebral and Cardiovascular Center 5‐7‐1, Fujishiro‐dai Suita Osaka 565‐8565 Japan

**Keywords:** Dental services, epidemiology, gerontology, mastication, oral hygiene, periodontal disease

## Abstract

There are numerous reports on the relationship between regular utilization of dental care services and oral health, but most are based on questionnaires and subjective evaluation. Few have objectively evaluated masticatory performance and its relationship to utilization of dental care services. The purpose of this study was to identify the effect of regular utilization of dental services on masticatory performance. The subjects consisted of 1804 general residents of Suita City, Osaka Prefecture (760 men and 1044 women, mean age 66.5 ± 7.9 years). Regular utilization of dental services and oral hygiene habits (frequency of toothbrushing and use of interdental aids) was surveyed, and periodontal status, occlusal support, and masticatory performance were measured. Masticatory performance was evaluated by a chewing test using gummy jelly. The correlation between age, sex, regular dental utilization, oral hygiene habits, periodontal status or occlusal support, and masticatory performance was analyzed using Spearman's correlation test and *t*‐test. In addition, multiple linear regression analysis was carried out to investigate the relationship of regular dental utilization with masticatory performance after controlling for other factors. Masticatory performance was significantly correlated to age when using Spearman's correlation test, and to regular dental utilization, periodontal status, or occlusal support with *t*‐test. Multiple linear regression analysis showed that regular utilization of dental services was significantly related to masticatory performance even after adjusting for age, sex, oral hygiene habits, periodontal status, and occlusal support (standardized partial regression coefficient *β* = 0.055). These findings suggested that the regular utilization of dental care services is an important factor influencing masticatory performance in a Japanese urban population.

## Introduction

Reducing the gap between average life span and healthy life expectancy has been identified as one of the most important challenges in Japanese health policy. The mainstream of dental care is shifting from conventional dental care mainly treating dental caries and periodontal disease to maintaining good oral function. Previous studies have reported the relationship between oral function and healthy life expectancy (Nasu and Saito, 2006), and between masticatory ability and quality of life (Takata et al., 2006). Receiving dental preventive treatment at regular intervals is recognized to be effective in maintaining oral health (Locker, 2001), but the lower rate of regular utilization in Japan versus that in the USA and Europe has been a long‐standing issue (Ikebe et al., 2002; McGrath et al., 1999). Within this context, the Healthy Japan 21 launched in 2000 to promote national health in the 21st century is attempting to promote regular dental service utilization as a national policy by targeting “a regular utilization of dental services rate of 30% or more” (Japan health promotion and fitness foundation, 2000).

Previous studies reported that sex (Astrom et al., 2013; Ikebe et al., 2002), age, economic prosperity (Donaldson et al., 2008; Ikebe et al., 2002; Stahlnacle et al., 2005), and educational background (Stahlnacle et al., 2005) were associated with regular dental utilization. In addition, the oral health status such as number of remaining teeth (Astrom et al., 2013; Donaldson et al., 2008) and periodontal status (Lang et al., 1994) have also been reported as related factors.

Many previous studies have used questionnaire‐based subjective evaluation; however, virtually no study has comprehensively examined the relationship of regular dental utilization to oral health status and oral function while including objective indicators such as masticatory performance. The aim of the study, therefore, was to investigate the relationship between regular dental utilization and masticatory performance through a cross‐sectional analysis in a general urban Japanese population.

## Materials and Methods

### Subjects

The subjects of this study were 1804 members of the general population (760 men and 1044 women; mean age of 66.5 ± 7.9 years) residing in Suita City, Osaka Prefecture, who underwent a health checkup as part of the Suita study, a prospective cohort study of cardiovascular diseases, implemented by the Department of Preventive Cardiology of the National Cerebral and Cardiovascular Center between June 2008 and July 2013 (Kokubo et al., 2008). A total of 2004 subjects was subjected to dental examination between June 2008 and July 2013. Of these, 200 individuals were excluded: 48 had no data on dental health behavior, 118 with incomplete periodontal data, and 34 incomplete masticatory performance data. Finally, 1804 subjects were included in the study. The present study was conducted with the approval of the Ethics Committee of National Cerebral and Cardiovascular Center (M19‐062‐4). All subjects gave written informed consent for their participation.

### Dental health behavior

Subjects were interviewed by dentists to determine whether they visited their dentist regularly, regardless of the presence or absence of subjective symptoms. In our study, visiting dentist at regular intervals regardless of the presence or absence of subjective symptoms is defined as “regular dental utilization.” A questionnaire was used to investigate the frequency of toothbrushing per day and whether or not interdental aids such as dental floss and interdental brushes were used. Based on frequency of toothbrushing, subjects were divided into two groups: toothbrushing <2 times and ≥2 times a day (Rimondini et al., 2001), and based on use of interdental aids, they were divided into two groups of yes or no.

### Posterior occlusal supports

Posterior occlusal contacts were recorded according to the Eichner Index (Eichner, 1990). This index is based on the number of occlusal contacts of existing natural teeth or fixed partial dentures between maxilla and mandible bilaterally in the premolar and molar regions. Group A has contacts in four support zones; group B has contact in one to three zones or in the anterior area only; and group C has no support zone at all, although a few teeth may still remain. The classification provides a standard for the degree of morbidity of dentition and is suitable for application in studies on morbidity statistics (Eichner, 1990; Ikebe et al., 2010).

### Periodontal status

Periodontal status was evaluated using the Community Periodontal Index (CPI) (Ainamo et al., 1982). The indexed teeth were the left and right maxillaries and mandibular first and second molars, maxillary right central incisor, and mandibular left central incisor (a total of 10 teeth) (Shirone et al., 2007). If the examination could not be performed because the central incisor was missing, the corresponding tooth on the other side was examined. Five dentists, who were calibrated prior to commencement of the study, measured six sites on each tooth using a CPI probe (YDM, Tokyo, Japan) based on the following criteria and recorded the highest code value. The CPI codes were as follows: Code 0, no signs of inflammation of the gingiva; Code 1, bleeding was evident after probing; Code 2, dental calculus deposits (including those detected by probing up to 4 mm beneath the gingival margin); Code 3, periodontal pocket of depth ≥4 mm but <6 mm; Code 4, periodontal pocket of depth ≥6 mm. In our study, periodontitis was defined as CPI Code ≥3.

### Masticatory performance

Masticatory performance was determined from the concentration of dissolved glucose obtained from test gummy jelly (20 × 20 × 10 mm, 5.5 g, UHA Mikakuto Co., Ltd., Osaka, Japan) that is a standardized food developed for measuring masticatory performance (Ikebe et al., 2005; Okiyama et al., 2003). Subjects were instructed to chew jelly using 30 chewing strokes on their preferred chewing side (right, left, or both sides), and to expectorate the bolus of comminuted particles as thoroughly as possible. The cotton gauze and comminuted particles were then rinsed under running water for 30 sec in order to remove saliva and glucose adhering to their surfaces. The comminuted jelly alone was subsequently placed in a plastic container, water (35°C, 15 mL) was injected into this container, and the contents were agitated for 10 sec with a magnetic stirrer (PC‐410D Digital Stirrer, Corning Incorporated, Tewksbury, MA, USA) (400 rpm). Immediately after this agitation, a small amount of the supernatant was collected using a set of forceps and placed in contact with the tip of a sensor fitted to a commercially available instrument for self‐monitoring blood glucose (Glutest Every, Sanwa Chemical Laboratory Co., Nagoya, Japan), and the glucose concentration (mg/dL) displayed after 15 sec was recorded. The increase in surface area of the comminuted jelly (in units of square millimeter) was calculated from the glucose concentration using a regression formula (*y* = 15*x* − 250), and this was regarded as masticatory performance (Okiyama et al., 2003). Participants who wore removable prostheses kept their dentures in place during measurement.

### Statistical analysis

The data analyses were performed using SPSS version 21.0 for Windows (SPSS Japan Inc., IBM Corporation, Tokyo, Japan). *P*‐values < 0.05 were considered statistically significant.

First, a chi‐squared test and *t*‐test were used to investigate whether differences in having regular dental utilization led to differences in various factors. The chi‐squared test was used for analyzing sex, brushing habits, flossing, Eichner classification, and periodontal status, and *t*‐test was used for age and masticatory performance.

Second, Spearman correlation coefficient, *t*‐test, and one‐way analysis of variance (ANOVA) were used to examine differences in masticatory performance with regard to each of the individual explanatory variables. Spearman correlation coefficient was used to evaluate the relationship between age and masticatory performance. *t*‐test was used for analyzing sex, regular dental utilization, brushing habits, flossing, and periodontal status, and one‐way ANOVA was used for Eichner classification.

Finally, multiple linear regression analysis was carried out to test the relationship of each explanatory variable with masticatory performance after controlling for other factors. The explanatory variable of age was left as a continuous variable. Regular dental utilization was scored as no = 0 and yes = 1. Periodontal status was scored as CPI 0–2 = 0 and CPI 3–4 = 1. Occlusal contact had three categories, from which dummy variables were created. In the regression analysis, Eichner group A was the reference category. All the explanatory variables were entered into the model.

## Results

About half of the participants had regular utilization of dental care services; women were significantly more likely to visit their dentist regularly (Table 1). There was statistically significant difference in percentage of brushing at least twice/day and using interdental aids by regular dental utilization (*P* < 0.001). Subjects with regular utilization of dental care services had significantly more occlusal support (*P* < 0.001) and better periodontal status (*P* = 0.049).

**Table 1:** Characteristics of study population and examination results based on regular dental utilization.

	Regular dental utilization		*P*‐value
	−	+	
	(*n* = 922)	(*n* = 882)	
Age	65.7 ± 8.0	67.3 ± 7.7	<0.001^a^
Men (%)	435 (47.2)	325 (36.8)	<0.001^b^
Brushing habit (%)	71.6	85.3	<0.001^b^
Flossing (%)	44.0	71.4	<0.001^b^
Eichner classification (%)			
A	60.6	66.1	<0.001^b^
B	30.7	30.0	–
C	8.7	3.9	–
CPI 3 or 4 (%)	52.7	48.0	0.049^b^
Masticatory performance (mm^2^)	4387 ± 1870	4677 ± 1812	<0.001^a^

Mean ± SD

Brushing habits, brushing more than two times/day.

CPI; Community Periodontal Index.

a *t*‐test was used for continuous variable.

b Chi‐squared test was used for categorical variable.

Masticatory performance was significantly associated with age, regular utilization of dental care services, occlusal support, and periodontal status, but not with sex (*P* = 0.067), brushing habits (*P* = 0.536), or flossing (*P* = 0.059) (Tables 2 and 3). Younger persons, subjects with regular utilization of dental care services, and good periodontal status had significantly higher masticatory performance than their counterparts. Participants in the Eichner A group had the highest mean masticatory performance among the three groups.

**Table 2:** Spearman correlation coefficients between masticatory performance and age.

	*r*s	*P*‐value
Age	−0.195	<0.001

*r*s, Spearman correlation coefficient.

**Table 3:** Bivariate analysis of masticatory performance (mm^2^) in relation to explanatory variables.

	Number of subjects	Mean ± SD	*P*‐value
Sex			
Men	760	4622 ± 1972	0.067^a^
Women	1044	4461 ± 1748	–
Regular dental utilization			
Yes	882	4677 ± 1812	<0.001^a^
No	922	4387 ± 1870	–
Brushing habits			
Yes	1412	4543 ± 1817	0.536^a^
No	392	4478 ± 1951	–
Flossing			
Yes	1036	4500 ± 1824	0.059^a^
No	768	4434 ± 1874	–
Eichner classification			
A	1142	5177 ± 1646	<0.001^b^
B	548	3728 ± 1669	–
C	114	2488 ± 1669	–
CPI 0, 1, or 2	895	4705 ± 1865	<0.001^a^
3 or 4	909	4356 ± 1813	–

Brushing habit, brushing more than two times/day.

CPI; Community Periodontal Index.

a *t*‐test was used for categorical variable.

b One‐way analysis of variance test was used for categorical variable.

The multiple linear regression analysis showed that, with other variables controlled, masticatory performance was significantly associated with age (*β*: standardized partial regression coefficient = −0.063), sex (*β* = −0.085), regular utilization of dental care services (*β* = 0.055), posterior occlusal support (*β* = −0.326 for Eichner B and *β* = −0.329 for Eichner C), and periodontal status (*β* = −0.084) (Table 4). Oral hygiene habits such as brushing habits and flossing ware not significant independent variables of masticatory performance.

**Table 4:** Multiple linear regression analysis with masticatory performance as the dependent variable and age, sex, regular dental utilization, brushing habits, flossing, occlusal support, and periodontal status as the independent variables.

Independent variable	*B*	*SE*	Beta	*P*‐value
Age	−14.9	5.2	−0.063	0.005
Sex	−317.1	82.7	−0.085	<0.001
Regular dental utilization	202.2	81.9	0.055	0.014
Brushing habits	16.1	98.8	0.004	0.871
Flossing	−3.5	82.9	−0.001	0.967
Occlusal support: Eichner A as a reference				
Eichner B	−1310.6	88.8	−0.326	<0.001
Eichner C	−2492.9	167.6	−0.329	<0.001
Community Periodontal Index	−311.0	78.5	−0.084	<0.001

Multiple *r* = 0.465; *r* ^2^ = 0.216; *P* < 0.05.

Dependent variable is masticatory performance.

Beta means standardized partial regression coefficient.

Age was used as a continuous variable.

Sex: men = 0, women = 1

Regular dental utilization: no = 0, yes = 1

Brushing habits: <2 times a day = 0, ≥2 times a day = 1

Flossing: no = 0, yes = 1

Eichner group A is the reference category.

Community Periodontal Index (CPI): CPI 0–2 = 0, CPI 3–4 = 1

## Discussion

To our knowledge, this is the first large‐scale study to show a relationship between regular utilization of dental care services and masticatory performance as the oral function indicator in general population. In the present study, subjects who utilized dental care services regularly had good oral hygiene habits, good periodontal status, and well‐maintained occlusal support compared with those who did not. In addition, the results of multiple regression analysis suggested that regular utilization of dental care services was significantly associated with masticatory performance after controlling periodontal status and occlusal support but oral hygiene habits were not significantly associated with masticatory performance. These findings suggest that regular utilization of dental care services has stronger association with masticatory performance than an individual's oral hygiene habits, and utilization of dental care services is very important to maintain masticatory performance.

In this study, periodontal status was evaluated using CPI. CPI is a simple method with a high level of repeatability used to measure only the indexed teeth. Because this method could be performed in a short time, it is suitable for evaluating a large number of subjects, and inter‐examiner agreement among the five dentists was good (Cohen's *κ* = 0.78). CPI based on partial examination cannot be used to evaluate all remaining teeth, but it is capable of screening approximately 85% of periodontitis patients relative to the CPI full examination method (Shirone et al., 2007), and this method is thought to be a valid approach for mass examination given the associated time restraints.

We measured the masticatory performance with test gummy jelly. Masticatory performance is usually assessed by measuring the size of test food samples that have been chewed for a specific number of chewing cycles. However, it is reported that masticatory performance values assessed by calculating the surface area of gelatin particles provide a wider range of measurement than the sieving method (Ikebe et al., 2012). Thus, it is possible to differentiate between various subjects' results better. This method using test gummy jelly can be performed with a high degree of accuracy and repeatability, by strictly regulating the operating temperature and time (Ikebe et al., 2005). Therefore, many previous epidemiological studies used test gummy jelly (Ikebe et al., 2012).

Utilization of dental care services is thought to consist of two aspects. The first is when individuals visit their dentist after becoming aware of pain or dysfunction, and the second is when individuals visit to prevent the onset of disease or for post‐treatment maintenance (Ohi et al., 2009). Atchison *et al*. (1993) reported the need to examine factors associated with the utilization of dental services for preventive care and to compare them with factors associated with illness‐related dental utilization of these services, suggesting that these two types display different health behaviors. The present study examined regular utilization of dental care services for the purpose of prevention without subjective symptoms in order to investigate the effect of regular utilization on masticatory performance.

In our study, we found that as much as 48.9% of subjects underwent regular utilization compared with the previous studies (32.8% and 18.0%) (Ikebe et al., 2002; Ohi et al., 2009). This is because the residents of Suita City, Osaka Prefecture, targeted by this study exceed the national and prefectural means in terms of living standard (Financial condition of Suita City, n.d.), suggesting that the high rate of utilization of dental care services is linked to economic prosperity. Moreover, the percentage of those who brushed at least twice a day was high among those who underwent regular utilization of dental care services, and there was a large difference in the use of interdental aids, indicating that these subjects possess good oral hygiene habits. This finding was attributed to the fact that subjects receive instructions on cleaning techniques and devices when attending regular utilization.

It is well known that high masticatory performance is achieved through coordination of biological factors including occlusal support, periodontal status, the temporomandibular joint, masticatory muscles, and higher brain function controlling peripheral masticatory organs. Posterior occlusal contact of the remaining dentition has been reported as a key predictor of reduction of masticatory performance in earlier studies (Ikebe et al., 2006), and this finding was confirmed in the present study. In addition, periodontal status has been reported as a factor that affects masticatory performance along with occlusal support and occlusal force in previous studies (Kosaka et al., 2014). In this study, regular utilization of dental care services was significantly associated with masticatory performance when adjusted for occlusal support and periodontal status. This result suggested that utilization of dental care services helps to detect any problem in subjects' mouth and treat them in early stages; therefore, it is possible to maintain high masticatory ability.

Low coefficient of determination of the multiple regression model (*r* = 0.465, *r* ^2^ = 0.216) was one of the limitations of this study. Measuring masticatory performance with test food is thought to be influenced by subjects' eating habits and behavior. *Ow et al*. (1998) reported that mandibular movement velocity during mastication was associated with masticatory performance and suggested that faster mandibular movement led to higher masticatory performance. Thus, there are many other factors that influence masticatory performance; we cannot clearly predict masticatory performance using the items surveyed in our study. However, our findings that not only maintaining occlusal support or good periodontal status but also utilizing dental care services is an important factor that influences masticatory performance may constitute a valuable finding for improving utilization of dental care services at a national level. In addition, sex was not significantly associated with masticatory performance in the bivariate analysis but strongly significant in the multiple regression analysis in our study.

This result might be related to the high prevalence of periodontal disease in men than women. The periodontal status was associated with masticatory performance in the Suita study (Kosaka et al., 2014). Therefore, we suggested that sex is not significant in the bivariate analysis influenced by high prevalence of periodontal disease in men, but strongly significant in the multiple regression analysis after adjusting for periodontal disease.

Another limitation of this study is that we did not investigate the content and interval of maintenance being performed during the regular utilization of dental care services. In general, when individuals visit their dentists for regular checkup, they undergo a check for dental disease as well as scaling, mechanical tooth cleaning, and in the case of denture wearers, adjustment of their dentures. It is impossible to deny that the content of each visit may differ depending on the individual and the dental clinic.

In addition, due to its cross‐sectional design, the present study failed to confirm causal relationships between regular utilization of dental care services and masticatory performance. In the future, longitudinal studies will be required to investigate whether utilization of dental care services improves oral hygiene habits, maintenance of good periodontal status, and occlusal support, and, to examine the relationship between these outcomes and maintenance of high masticatory performance.

## Conclusions

The present study showed that general urban residents who visited their dentist for regular utilization had good oral hygiene habits, good periodontal status, and well‐maintained occlusal support compared with those who did not. Our findings suggest that not only maintaining occlusal support or good periodontal status but also utilizing dental care services may guarantee good masticatory performance.

## Conflicts of Interest

The authors declare no potential conflict of interest.

## References

[cre210-bib-0001] Ainamo, J. , Barmes, D. , Beagrie, G. , Cutress, T. , Martin, J. , Sardo‐Infirri, J. , 1982 Development of the World Health Organization (WHO) Community Periodontal Index of Treatment Needs (CPITN). Int. Dent. J. 32, 281–291. 6958657

[cre210-bib-0002] Astrom, A.K. , Ekback, G. , Nasir, E. , Ordell, S. , Unell, L. , 2013 Use of dental services throughout middle and early old ages: a prospective cohort study. Community Dent. Oral Epidemiol. 41, 30–39. 22937755 10.1111/j.1600-0528.2012.00709.x

[cre210-bib-0003] Atchison, K.A. , Mayer‐Oakes, S.A. , Schweitzer, S.O. , Lubben, J.E. , De Jong, F.J. , Matthias, R.E. , 1993 The relationship between dental utilization and preventive participation among a well‐elderly sample. J. Public Health Dent. 53, 88–95. 8515416 10.1111/j.1752-7325.1993.tb02681.x

[cre210-bib-0004] Donaldson, A.N. , Everitt, B. , Newton, T. , Steele, J. , Sheriff, M. , Bower, E. , 2008 The effect of social class and dental attendance on oral health. J. Dent. Res. 87, 60–64. 18096895 10.1177/154405910808700110

[cre210-bib-0005] Eichner, K. , 1990 Renewed examination of the group classification of partially edentulous arches by Eichner and application advices for studies on morbidity statistics. Stomatol. DDR 40, 321–325. 2270610

[cre210-bib-0006] Financial condition of Suita City . http://www.city.suita.osaka.jp/library/kobo/seisaku/page/004583/upload/zaiseijyoukyou.pdf [last accessed 9 June 2015]

[cre210-bib-0007] Ikebe, K. , Nokubi, T. , Ettinger, R.L. , 2002 Utilization of dental health services by community‐dwelling older adults in Japan who attended a weekly educational programme. Gerodontology 19, 115–122. 12542221 10.1111/j.1741-2358.2002.00115.x

[cre210-bib-0008] Ikebe, K. , Morii, K. , Matsuda, K. , Hazeyama, T. , Nokubi, T. , 2005 Reproducibility and accuracy in measuring masticatory performance using test gummy jelly. Prosthodontic. Res. Pract. 4, 9–15.

[cre210-bib-0009] Ikebe, K. , Matsuda, K. , Morii, K. , Furuya‐Yoshinaka, M. , Nokubi, T. , Renner, R.P. , 2006 Association of masticatory performance with age, posterior occlusal contacts, occlusal force, and salivary flow in older adults. Int. J. Prosthodontics. 19, 475–481. 17323726

[cre210-bib-0010] Ikebe, K. , Matsuda, K. , Murai, S. , Maeda, Y. , 2010 Validation of the Eichner index in relation to occlusal force and masticatory performance. Int. J. Prosthodont. 23, 521–524. 21209986

[cre210-bib-0011] Ikebe, K. , Matsuda, K. , Kagawa, R. , Enoki, K. , Okada, T. , Yoshida, M. , Maeda, Y. , 2012 Masticatory performance in older subjects with varying degrees of tooth loss. J. Dent. 40, 71–76. 22037296 10.1016/j.jdent.2011.10.007

[cre210-bib-0012] Japan health promotion and fitness foundation 2000 http://www.mhlw.go.jp/file/06‐Seisakujouhou‐10900000‐Kenkoukyouku/0000047330.pdf. [last accessed 9 June 2015].

[cre210-bib-0013] Kokubo, Y. , Kamiden, K. , Okamura, T. , Watanabe, M. , Higashiyama, A. , Kawanishi, K. et al., 2008 Impact of high‐normal blood pressure on the risk of cardiovascular disease in a Japanese urban cohort: the Suita study. Hypertension 52, 652–659. 18725580 10.1161/HYPERTENSIONAHA.108.118273

[cre210-bib-0014] Kosaka, T. , Ono, T. , Yoshimuta, Y. , Kida, M. , Kikui, M. , Nokubi, T. et al., 2014 The effect of periodontal status and occlusal support on masticatory performance: the Suita study. J. Clin. Periodontol. 41, 497–503. 24527750 10.1111/jcpe.12241

[cre210-bib-0015] Lang, W.P. , Farghaly, M.M. , Ronis, D.L. , 1994 The relation of preventive dental behaviors to periodontal health status. J. Clin. Periodontol. 21, 194–198. 8157773 10.1111/j.1600-051x.1994.tb00303.x

[cre210-bib-0016] Locker, D. , 2001 Does dental care improve the oral health of older adults? Community Dent. Health 18, 7–15. 11421409

[cre210-bib-0017] McGrath, C. , Bedi, R. , Dhawan, N. , 1999 Factors influencing older people's self‐reported use of dental services in the UK. Gerodontology 16, 97–102. 10825848 10.1111/j.1741-2358.1999.00097.x

[cre210-bib-0018] Nasu, I. , Saito, Y. , 2006 Active life expectancy for elderly Japanese by chewing ability. Jpn. J. Public Health 53, 411–423. 16881529

[cre210-bib-0019] Ohi, T. , Sai, M. , Kikuchi, M. , Hattori, Y. , Tsuboi, A. , Hozawa, A. et al., 2009 Determinants of the utilization of dental services in a community‐dwelling elderly Japanese population. Tohoku J. Exp. Med. 218, 241–249. 19561395 10.1620/tjem.218.241

[cre210-bib-0020] Okiyama, S. , Ikebe, K. , Nokubi, T. , 2003 Association between masticatory performance and maximal occlusal force in young men. J. Oral Rehabil. 30, 278–282. 12588500 10.1046/j.1365-2842.2003.01009.x

[cre210-bib-0021] Ow, R.K. , Carlsson, G.E. , Karlsson, S. , 1998 Relationship of masticatory movements to masticatory performance of dentate adults: a method study. J. Oral Rehabil. 25, 821–829. 9846902 10.1046/j.1365-2842.1998.00325.x

[cre210-bib-0022] Rimondini, L. , Zolfanelli, B. , Bernardi, F. , Bez, C. , 2001 Self‐preventive oral behavior in an Italian university student population. J. Clin. Periodontol. 28, 207–211. 11284532 10.1034/j.1600-051x.2001.028003207.x

[cre210-bib-0023] Shirone, K. , Ogawa, H. , Hirotomi, T. , Takano, N. , Yamaga, T. , Kaneko, N. et al., 2007 Evaluation of CPI and loss of attachment scoring methods (WHO) and longitudinal study on periodontal conditions in Japanese elderly cohort. J. Dent. Hlth. 57, 28–35.

[cre210-bib-0024] Stahlnacle, K. , Soderfeldt, B. , Unell, L. , Halling, A. , Axtelius, B. , 2005 Changes over 5 years in utilization of dental care by a Swedish age cohort. Community Dent. Oral Epidemiol. 33, 64–73. 15642048 10.1111/j.1600-0528.2004.00198.x

[cre210-bib-0025] Takata, Y. , Ansai, T. , Awano, S. , Fukuhara, M. , Sonoki, K. , Wakisaka, M. et al., 2006 Chewing ability and quality of life in an 80‐year‐old population. J. Oral Rehabil. 33, 330–334. 16629890 10.1111/j.1365-2842.2005.01567.x

